# Neutrophils to lymphocytes ratio as a useful prognosticator for stage II colorectal cancer patients

**DOI:** 10.1186/s12885-018-5042-x

**Published:** 2018-12-03

**Authors:** Nikoletta Dimitriou, Evangelos Felekouras, Ioannis Karavokyros, Andreas Alexandrou, Emmanuel Pikoulis, John Griniatsos

**Affiliations:** Department of Surgery, National and Kapodistrian University of Athens, Medical School, Laiko Hospital, Agiou Thoma 17 str, GR 115-27 Athens, Greece

**Keywords:** Colorectal cancer, Prognosis, Inflammatory markers, Neutrophil to lymphocyte ratio, Survival

## Abstract

**Background:**

The incidence of colorectal cancer (CRC) is expected to increase by 80% in year 2035. Even though advantages in treatment of CRC have being made over the last decades, the outcome remains poor. Recently, several inflammatory markers including pretreatment neutrophil to lymphocyte ratio (NLR), have being used as prognostic factors, since host inflammatory response to cancer is believed to determine disease progression.

The aim of this study is to evaluate the prognostic significance of pretreatment NLR, in terms of overall survival (OS), 5-year survival, disease-free survival (DFS) and recurrence, in CRC patients who underwent curative resection.

**Methods:**

We retrospectively reviewed 296 patients, who were submitted to elective surgery as first therapeutic option in curative intent, between January 2010 and December 2015. Pretreatment NLR, as well as demographics, clinical, histopathologic, and laboratory data were analyzed. Univariate and multivariate analyses were conducted to identify prognostic factors associated with OS, 5-year survival, DFS and recurrence.

**Results:**

The cutoff point of NLR was calculated with Kaplan-Meier curves and log-rank test to 4.7. Univariate and multivariate analyses disclosed elevated NLR as a significant dismal prognostic factor for DFS (HR 1.88; 95% CI 1.01–3.52; *p* = 0.048), 5-year survival (HR 2.14; 95% CI 1.12–4.10; *p* = 0.021) and OS (HR 2.11; 95% CI 1.11–4.03; *p* = 0.023). In a subgroup analysis, in patients with stage II CRC, NLR > 4.7 was a stronger poor predictor for DFS (HR 2.76; 95% CI 1.07–7.13; *p* = 0.036), 5-year survival (HR 3.84; 95% CI 1.39–10.63; *p* = 0.01) and OS (HR 3.62; 95% CI 1.33–4.82; *p* = 0.012). After adjusting stage for gender, age, location of the primary tumor, differentiation, as well as the presence of perineural, vascular, and lymphovascular invasion, the significance of NLR > 4.7 became more prominent for DFS (HR 2.85; 95% CI 1.21–6.73; *p* = 0.0176), 5-year survival (HR 4.06; 95% CI 1.66–9.93; *p* = 0.002) and OS (HR 4.07; 95% CI 1.69–9.91; *p* = 0.002) in stage II patients.

**Conclusion:**

Pretreatment NLR > 4.7 is a poor prognostic factor for DFS, 5-year survival and OS in CRC patients undergoing curative resection. The dismal prognostic effect of NRL is magnified in Stage II CRC patients.

## Background

The “classical model” of colorectal carcinogenesis is from normal mucosa to adenoma, then to dyspasia and then to cancer, the so-called “adenoma-carcinoma sequence” [1]. The molecular sequence of the above events include early loss of regulation of the Wnt signaling pathway, accumulation of activating mutations in oncogenes such as KRAS and BRAF, mutations in TP53 and SMAD4 genes and chromosomal instability, finally leading to malignant transformation [2].

Inflammation is currently considered as a hallmark for cancer development [3]. Current evidence addresses that within a tumor tissue and beside cancer cells, host structures (e.g. extracellular matrix), non-immune cells (e.g. fibrous tissue cells) and immune cells namely eosinophils, basophils, mast cells, lymphocytes, natural killer cells and dendritic cells [4–6], interact and contribute to a highly immunosuppressive microenvironment [7]. Lymphocytes have a crucial role in this microenvironment, since progressive increase in tumor-infiltrating lymphocytes is directly correlated with antitumor activity [8]. Intratumoral inflammation is assessed in hematoxylin and eosin stained tissue sections of formalin-fixed paraffin-embedded tumor specimens, scoring the average of chronic inflammation cells density within neoplastic cells’ area.

On the other hand, tissue hypoxia and necrosis [9] can cause complex interactions between the tumor and the nonspecific host inflammatory response, finally favoring disease’s progression in cancer [10]. That systemic inflammatory response involves alterations in neuroendocrine and hematopoietic system, in protein and energy metabolism and in liver function. Hepatocytes synthesize and release into the systemic circulation acute-phase proteins which are associated with lymphocytopenia and impaired T lymphocytic response within the tumor, compromising cell-mediated immunity [11]. A dysregulated systemic inflammatory response promotes cancer progression [12], while the presence of a systemic inflammatory response is associated with reduced survival [11]. For systemic inflammatory response estimation, serum levels of white blood cells, neutrophils, lymphocytes, platelets, C-reactive protein (CRP) and albumin, either alone or in several combinations, have been used as prognostic factors in various malignant solid tumors.

Roxburgh et al [13] disclosed that intratumoral and systemic inflammation are linked through the cell-mediated immune system, further stating that the type, density and location of a variety of immune cells, and not an individual immune cell type, are the important independent determinants of cancer-specific survival in patients with colorectal cancer [14], while Turner et al [15] demonstrated that intratumoral and systemic inflammatory responses appeared to be largely independent of each other.

Comparative analyses of the two inflammatory responses disclosed that when a high-grade intratumoral immune response exists, the longer the survival [14], high tumor infiltration by chronic inflammatory cells combined with low systemic inflammation are correlated to a significantly better prognosis [15], while increased systemic inflammatory response is consistently associated with a poor outcome independently to the tumor stage [16, 17]. Hence, a decreased intratumoral inflammation response and an increased systemic one might indicate a decreased immunological local control of the tumor, producing a systemic pro-inflammatory environment which facilitates cancer progression [4, 18].

NLR has been proposed as reflecting the balance between pro-tumor inflammation and anti-tumor immune function [19] and its prognostic significance has been extensively studied in several solid tumors [20]. Pronounced intratumoral lymphocytic infiltration has been proposed as a novel independent prognostic factor for colorectal cancer patients, even superior to the Dukes’ staging system [21], while systemic inflammation response may serve as a supplemental index in TNM staging system [22, 23].

The aim of this study was to evaluate the prognostic significance of pretreatment NLR, in terms of overall survival (OS), 5-year survival, disease-free survival (DFS) and recurrence in CRC patients, who underwent curative resection, without neoadjuvant treatment.

## Methods

### Patients

From 2009 onwards, all patients who were referred to our Department for further investigation and treatment, having been diagnosed with colorectal tumors, were prospectively enrolled. Upon their admission, all patients were informed that their details such as demographics, clinical data, laboratory results, adjuvant or neo-adjuvant therapies, type of operation, postoperative complications, histological findings, follow-up, elapse time to either local or distant recurrence, short and long term outcome as well as survival, will be prospectively collected. All agreed to participate and all consented for free use of their details for scientific purposes (research, presentations, publications, etc) in the future.

Between January 2010 and December 2015, 360 patients suffering from colorectal cancer were submitted to surgery, as first therapeutic option in curative intent, in the 1st Department of Surgery of the University of Athens, in “Laiko” Hospital.

In accordance to the Declaration of Helsinki, the present study was approved by the Scientific Council of the “Laiko” Hospital.

All patients suffered from sporading colorectal cancer and all had undergone colonoscopy and biopsies for histological confirmation of the disease. For staging of the metastatic disease, they underwent at least computer tomography (CT) of thorax and abdomen. Patients with rectal cancer were further submitted to magnetic resonance imaging (MRI) of the pelvis for loco-regional disease staging [24].

Prior to any therapeutic option implementation, all cases were discussed in the Multi-Disciplinary Cancer meeting (which comprised Surgeons, Oncologists, Radiologists and Pathologists). The most suitable therapeutic strategy was planned and was adopted by all surgeons.

Excluding patients: (i) with uncompleted data (*n* = 5), (ii) who died within 90 days from the initial operation (*n* = 6), (iii) who were histologically classified as Tis (*n* = 35), (iv) who were diagnosed as stage IV, even though a curative resection was achieved (*n* = 8) and (vi) who suffered from multiple distant metastases (*n* = 10), a total of 296 adenocarcinoma patients were enrolled in the present study and retrospectively analyzed.

### Hematological tests

All blood samples were taken within three days before surgery. In this way, any kind of infection and co-existing inflammatory disease could be reliably excluded [25]. NLR was defined as the absolute neutrophil count divided by the absolute lymphocyte count.

### Optimal cut-off value for NLR

In literature, there is an increasing interest in finding the optimal threshold value above which NLR significantly increases the likelihood of death or recurrence [26–30].

This has been typically carried out using ROC curves, which visually represent the sensitivity (i.e. probability of correctly identifying an event e.g. a death) and the specificity (i.e. probability of correctly identifying a nonevent) of various cutoffs. However, main disadvantage of the method is that it cannot distinguish censored from fully observed survival times. Thus, patients who were lost during follow up, were remained throughout calculations, independently if their survival times were censored or not. Hence, failure to take into account the censored times can yield to misleading inferences. In the present study, we used a method [30] based on Kaplan-Meier curves and the logrank test, which do account for censoring. For a range of potential threshold values of NLR, we calculated the Kaplan-Meier curves and the logrank test, selecting the threshold giving the greatest separation of curves in terms of the lowest *p*-value.

### Oncologic outcome

The pathological stage of the disease was based on the 7th TNM Classification [31]. The elapse period from the initial operation to the development of the recurrence, the site and the organ of recurrence, the therapeutic strategies and the final outcome were documented during the follow-up, for DFS and OS estimation.

### Statistical analysis

Statistical analyses were performed using the STATA statistical package (Version 13.0, Stata Corp, College Station, Texas). Quantitative variables were summarized as median and Interquartile Range (IR) when deviation from normal distribution was observed. Histograms and distribution plots (Percentile-Percentile and Quantile-Quantile plots) were used to evaluate the normality of the quantitative variables. Categorical variables were summarized as absolute and percentage values. Such descriptive statistics were presented for the overall sample, as well as for the NLR category. For quantitative variables, *p*-values were based on the t-test or the Mann–Whitney U test, if non-normality was seen. Association between categorical variables was measured through Fisher’s exact test. The significance level was pre-determined at 5%.

We used standard Cox proportional hazards models, in order to study the effect of NLR on OS, 5-year survival, DFS and recurrence. Known significant demographic, clinical and tumor characteristics were taken into account to examine whether there is an independent association of NLR with the event of interest.

## Results

There were 114 female patients with a median age of 71 years (IR 63–79) and 182 male patients with a median age of 72 years (IR 63–77). The clinicopathologic characteristics of the enrolled patients are presented in Table 1.

**Table 1:** Clinicopathological characteristics of the enrolled patients

Parameter	No of patients (*n* = 296)	%
Neutrophil / Lymphocyte Ratio		
NLR ≤ 4.7	260	87.8
NLR > 4.7	36	12.2
Gender		
Female	114	38.5
Male	182	61.5
Age (years)		
Median + IR	72 (63–77)	
Age > 72		
Age ≤ 72	157	53
Age > 72	139	47
Primary tumor		
Right colon	103	34.8
Left colon	62	21
Rectum	131	44.2
Differentiation		
Low	63	21.3
Medium + High	233	78.7
Τ		
T1	18	6
T2	57	19.2
T3	195	65.9
T4	26	8.8
Ν		
N0	187	63.2
N1	77	26
N2	32	10.8
No of lymph nodes harvested		
Median + IR	19 (14–27.5)	
No of lymph nodes harvested		
Lymph nodes ≥12	261	88.2
Lymph nodes < 12	35	11.8
Stage		
Stage I	61	20.6
Stage II	126	42.6
Stage III	109	36.8
Perineural invasion		
No	273	92.2
Yes	23	7.8
Vascular invasion		
No	253	85.5
Yes	43	14.5
Lymphatic invasion		
No	257	86.8
Yes	39	13.2
Follow-up (months)		
Median + IR	45 (27–68.5)	
Site of recurrence		
Distant	16	51.6
Local	15	48.4
Follow up		
Alive with recurrence	8	2.7
Deaths related to the recurrence	23	7.8
Deaths unrelated to the disease	45	15.2
Alive	220	74.3

### Distribution of NLR values among several clinicopathological variables (Table 2)

The median value of NLR for the whole study population was 2.55 (IR: 1.93–3.41). Statistically significant increased median values of NLR was found among patients older than 72 years old compared to them younger than 72, and in primary tumors in which histology report disclosed perineural and vascular invasion. Moreover, there was a gradually increased median value of NLR from T1 to T4 tumors, (1.88, 2.49, 2.60 and 2.76, respectively) although a statistical significance was not reached under any possible combination. The lowest median NLR value was noticed in Stage I patients. However, a marginally statistically significant difference (*p = 0.049*) was noticed when stage I patients compared to the aggregated stage II and III patients. Although not statistically significant, we should mentioned that the highest median NLR values were detected among primary tumors positive for perineural invasion (3.45), among patients who developed distant metastases (3.05), among them who developed recurrence of the disease (2.79) and among N2 patients (2.78).

**Table 2:** NLR among several clinicopathological variables

Parameter	NLR (Median + IR)	*p value*
Gender		
Male	2.59 (2.05–3.32)	*NS*
Female	2.51 (1.87–3.69)	
Age		
≥ 72 years	2.72 (2.14–3.76)	*0.016*
< 72 years	2.41 (1.82–3.16)	
Primary tumor		
Right colon	2.67 (2.13–3.76)	
Left colon	2.65 (1.89–3.42)	
Rectum	2.48 (1.81–3.08)	
Right VS Left colon	2.67 (2.13–3.76) VS 2.49 (1.85–3.24)	*NS*
Colon VS Rectum	2.67 (2.10–3.68) VS 2.48 (1.81–3.08)	*NS*
Differentiation		
Low	2.7 (2.2–3.44)	*NS*
Medium + High	2.51 (1.88–3.4)	
T		
T1	1.88 (1.53–2.98)	*NS*
T2	2.49 (1.85–3.14)	
T3	2.6 (2.0–3.43)	
T4	2.76 (2.27–5.22)	
N		
N0	2.58 (1.92–3.37)	*NS*
N1	2.34 (1.85–3.45)	
N2	2.78 (2.29–3.95)	
No of lymph node harvested		
< 12	2.37 (2.09–3.28)	*NS*
≥ 12	2.58 (1.92–3.43)	
No of infiltrated lymph nodes		
0 (*n* = 198)	2.58 (1.96–3.43)	*NS*
1 (*n* = 21)	2.22 (1.73–3.82)	
≥ 2 (*n* = 77)	2.55 (1.91–3.33)	
Stage		
Stage I	2.26 (1.74–2.87)	*0.049*
Stage II	2.67 (2.16–3.45)	
Stage III	2.55 (1.96–3.60)	
Stage I VS Stage II + III	2.26 (1.74–2.87) VS 2.62 (2.07–3.48)	
Perineural invasion		
No	2.53 (1.91–3.30)	*< 0.001*
Yes	3.45 (2.24–6.00)	
Vascular invasion		
No	2.54 (1.91–3.36)	*0.034*
Yes	2.68 (2.17–4.18)	
Lymphatic invasion		
No	2.53 (1.91–3.40)	*NS*
Yes	2.63 (2.12–3.69)	
Recurrence		
No	2.54 (1.96–3.37)	*NS*
Yes	2.79 (1.83–4.03)	
Site of recurrence		
Local (*n* = 15)	2.11 (1.56–4.03)	*NS*
Distant (*n* = 16)	3.05 (2.07–3.89)	

### Correlation between NLR and several clinicopathological variables

Setting the NLR cut-off value at 4.7, patients were divided in two groups (NLR ≤ 4.7, *n* = 260 and NLR > 4.7, *n* = 36). Univariate analysis among several clinicopathological variables between the two groups (Table 3), revealed that patients with NLR > 4.7 were most likely of advanced age (*p = 0.004*), elderly than 72 years old (*p = 0.033*), with nearly doubled probability for disease-related death (*p = 0.012*) and worse overall survival (*p = 0.036)*, compared to the patients with NLR ≤ 4.7.

**Table 3:** Univariate analysis between the two groups of patients, after setting the NLR cutoff value at 4.7

Parameter	NLR ≤ 4.7 (*n* = 260)	NLR > 4.7 (*n* = 36)	*p-value*
Gender			
Female	96	18	*NS*
Male	164	18	
Age (years)			
Median + IR	71 (63–77)	75.5 (70.5–81)	*0.004*
Age > 72			
Age **≤** 72	144	13	*0.033*
Age > 72	116	23	
Primary tumor			
Right colon	88	15	*NS*
Left colon	54	8	
Rectum	118	13	
Differentiation			
Low	56	7	*NS*
Medium + High	204	29	
Τ			
T1	17	1	*NS*
T2	49	8	
T3	175	20	
T4	19	7	
Ν			
N0	165	22	*NS*
N1	68	9	
N2	27	5	
No of lymph nodes harvested			
Median + IR	19 (14–27)	17 (15–28)	*NS*
No of lymph nodes harvested			
Lymph nodes ≥12	230	31	*NS*
Lymph nodes < 12	30	5	
Stage			
Stage I	55	6	*NS*
Stage II	110	16	
Stage III	95	14	
Perineural invasion			
No	246	27	*< 0.001*
Yes	14	9	
Vascular invasion			
No	226	27	*NS*
Yes	34	9	
Lymphatic invasion			
No	227	30	*NS*
Yes	33	6	
Site of recurrence			
Distant	12	4	*NS*
Local	14	1	
Follow up			
Alive with recurrence	8	0	*0.036*
Deaths related to the recurrence	18	5	
Deaths unrelated to the disease	35	10	
Alive	199	21	
Probability for overall survival			
Alive	204	21	*0.012*
Dead	56	15	
Probability for recurrence			
No recurrence	234	31	*NS*
Recurrence	26	5	

### Oncologic outcome

Recurrence, DFS, 5-year survival and OS were set as the end points for the oncologic outcome of the patients.

Within a median follow up of 45 months (IR 27–68.5), 31 patients developed recurrence of the disease. Fifteen patients developed local recurrence, while the remaining 16 developed distant metastases namely: liver (*n* = 7), lung (*n* = 3), simultaneous lung and liver (*n* = 3) and disseminated peritoneal carcinomatosis (*n* = 3). Eleven patients who developed local recurrence and 12 patients who developed distant metastases, died from the disease.

Multivariate analyses among factors which might influence recurrence (Table 4) disclosed that stages II and III, as well as vascular invasion were independently related to a worse prognosis. However, NLR was unrelated to the recurrence.

**Table 4:** Univariate and Multivariate analysis among factors which might affect the recurrence and the disease free survival (DFS)

	Recurrence						DFS					
	Univariate Analysis			Multivariate Analysis			Univariate Analysis			Multivariate Analysis		
	HR	95% CI	*p value*	HR	95% CI	*p value*	HR	95% CI	*p value*	HR	95% CI	*p value*
NLR												
≤ 4.7	1			1			1			1		
> 4.7	1.67	0.64–4.36	*0.293*	1.1	0.38–3.21	*0.862*	2.03	1.16–3.57	*0.014*	1.88	1.01–3.52	*0.048*
Gender												
Female	1			1			1			1		
Male	1.81	0.81–4.04	*0.15*	1.73	0.76–3.93	*0.189*	1.36	0.84–2.18	*0.207*	1.41	0.87–2.30	*0.163*
Age												
≤ 72	1			1			1			1		
> 72	0.9	0.44–1.83	*0.763*	1.02	0.48–2.17	*0.949*	1.70	1.08–2.65	*0.021*	1.83	1.15–2.91	*0.011*
Primary tumor												
Right colon	1			1			1			1		
Left colon	0.84	0.29–2.46	*0.753*	0.7	0.23–2.14	*0.535*	0.81	0.41–1.61	*0.543*	0.71	0.35–1.46	*0.354*
Rectum	1.34	0.61–2.96	*0.463*	1.53	0.66–3.55	*0.316*	1.39	0.85–2.28	*0.196*	1.46	0.85–2.51	*0.175*
Grade												
Low	1			1			1			1		
Medium + High	1.43	0.55–3.73	*0.464*	1.98	0.73–5.35	*0.177*	1.14	0.65–2.00	*0.646*	1.18	0.66–2.14	*0.574*
T												
T1	1						1					
T2	8.74E + 08	8.74e + 08–8.74e + 08	*.*				2.96	0.38–22.96	*0.299*			
T3	1.68e + 09	5.79e + 08–4.88e + 09	*0*				5.2	0.72–37.60	*0.102*			
T4	3.33e + 09	8.89e + 08–1.25e + 10	*0*				8.95	1.16–69.40	*0.036*			
N												
N0	1						1					
N1	1.06	0.43–2.57	*0.906*				1.04	0.61–1.79	*0.884*			
N2	3.60	1.54–8.46	*0.003*				2.88	1.64–5.07	*< 0.001*			
No lymph nodes yield												
≥ 12	1						1			1		
< 12	0.55	0.13–2.29	*0.408*	0.55	0.13–2.44	*0.433*	1.56	0.86–2.83	*0.145*	1.7	0.89–3.24	*0.106*
Stage												
Stage I	1						1			1		
Stage II	3.86	0.88–16.99	*0.074*	5.14	1.14–23.30	*0.034*	2.35	1.09–5.07	*0.029*	2.80	1.28–6.10	*0.01*
Stage III	4.81	1.10–21.05	*0.037*	5.22	1.13–24.13	*0.035*	2.80	1.30–6.03	*0.008*	3.06	1.38–6.78	*0.006*
Perineural invasion												
No	1						1			1		
Yes	2.03	0.71–5.84	*0.189*	0.89	0.26–3.05	*0.848*	1.37	0.63–2.99	*0.428*	0.54	0.22–1.32	*0.176*
Vascular invasion												
No	1			1			1			1		
Yes	4.05	1.93–8.49	*0*	4.23	1.78–10.03	*0.001*	2.36	1.39–4.02	*0.001*	2.38	1.26–4.49	*0.007*
Lymphatic invasion												
No	1			1			1			1		
Yes	2.42	1.04–5.64	*0.04*	1.16	0.43–3.11	*0.769*	2.07	1.20–3.59	*0.009*	1.48	0.77–2.83	*0.237*

**Fig. 1:**
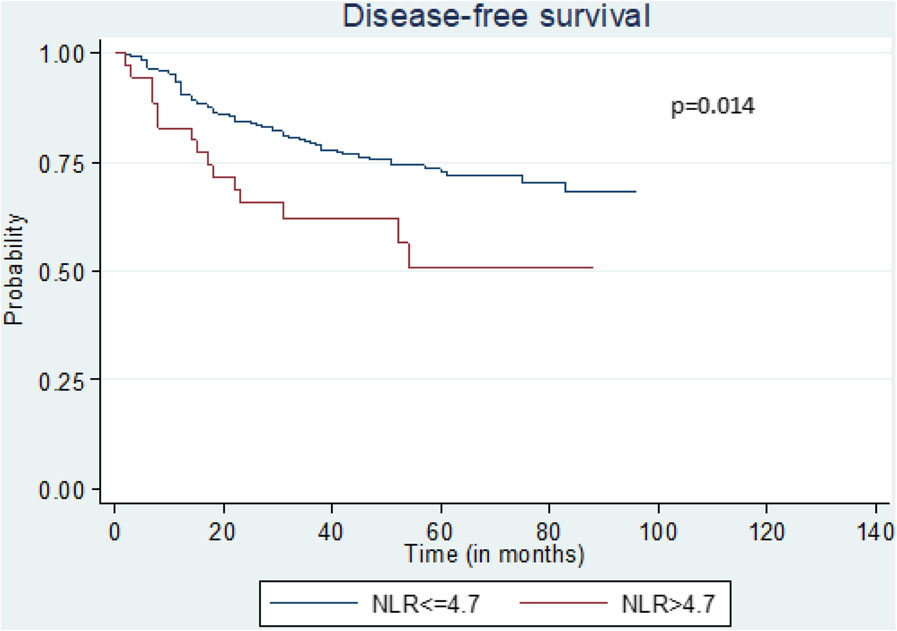
Kaplan-Meier plots quantifying the effects of NLR status on the DFS

**Fig. 2:**
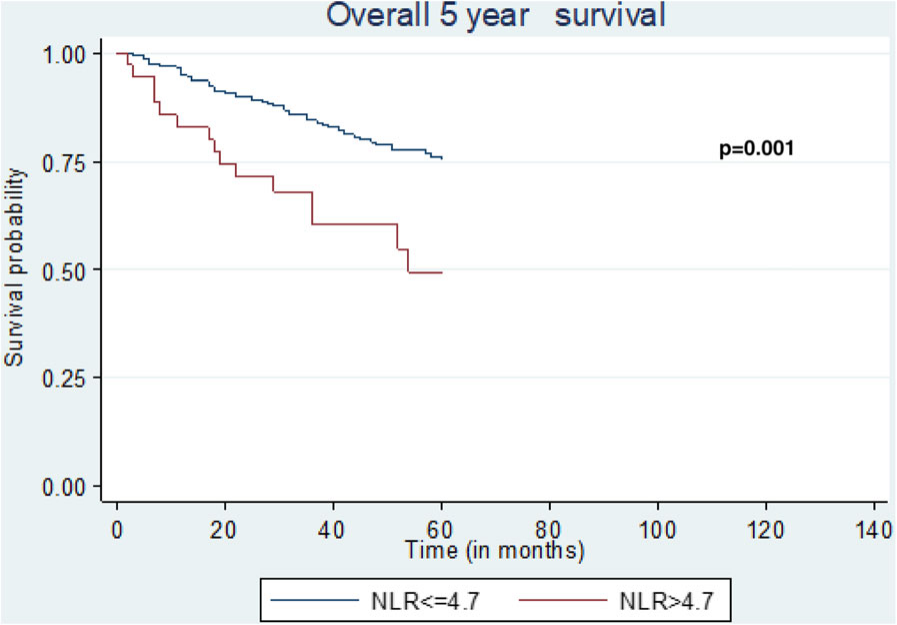
Kaplan-Meier plots quantifying the effects of NLR status on the 5-year survival

**Fig. 3:**
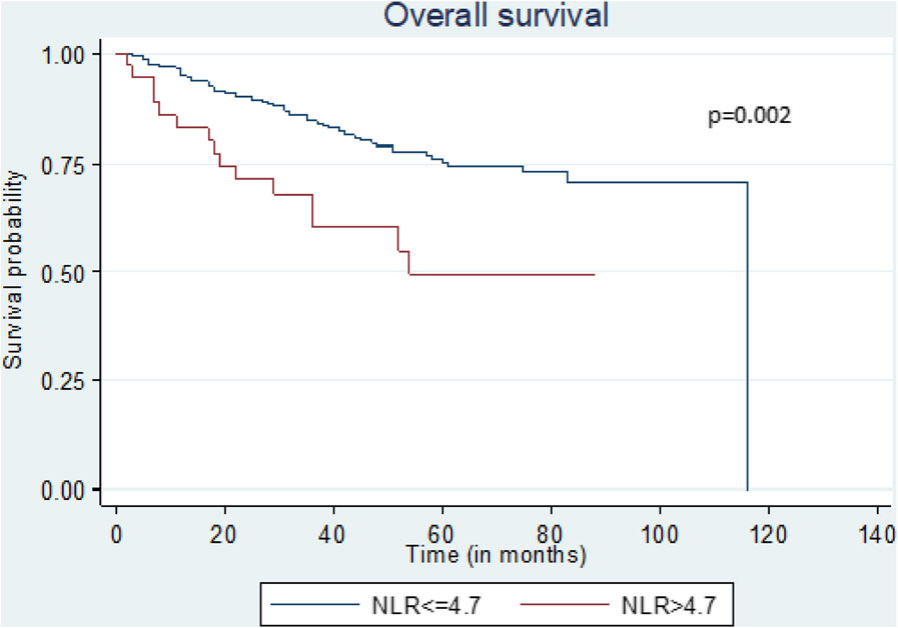
Kaplan-Meier plots quantifying the effects of NLR status on the overall survival

On the other hand (Tables 4 and 5), NLR > 4.7, age above 72 years-old, stages II and III and vascular invasion had independent adverse effect on DFS (Fig. 1), 5-year survival (Fig. 2) and OS (Fig. 3).

**Table 5:** Univariate and Multivariate analysis among factors which might affect the 5-year survival and the overall survival (OS)

	5-year survival						Overall survival					
	Univariate Analysis			Multivariate Analysis			Univariate Analysis			Multivariate Analysis		
	HR	95% CI	*p value*	HR	95% CI	*p value*	HR	95% CI	*p value*	HR	95% CI	*p value*
NLR												
≤ 4.7	1			1			1			1		
> 4.7	2.58	1.45–4.59	*0.001*	2.14	1.12–4.10	*0.021*	2.48	1.40–4.40	*0.002*	2.11	1.11–4.03	*0.023*
Gender												
Female	1			1			1			1		
Male	1.11	0.67–1.83	*0.681*	1.22	0.72–2.04	*0.459*	1.18	0.72–1.93	*0.517*	1.29	0.78–2.16	*0.324*
Age												
≤ 72	1			1			1			1		
> 72	2.05	1.25–3.35	*0.004*	2.30	1.37–3.87	*0.002*	2.13	1.32–3.46	*0.002*	2.41	1.45–4.01	*0.001*
Primary tumor												
Right colon	1			1			1			1		
Left colon	0.88	0.42–1.82	*0.73*	0.79	0.37–1.70	*0.552*	0.84	0.41–1.72	*0.627*	0.76	0.35–1.61	*0.467*
Rectum	1.35	0.79–2.32	*0.275*	1.38	0.76–2.50	*0.283*	1.35	0.80–2.29	*0.262*	1.37	0.77–2.44	*0.289*
Grade												
Low	1			1			1			1		
Medium + High	1	0.55–1.80	*0.995*	0.99	0.53–1.85	*0.98*	1.04	0.58–1.87	*0.891*	1	0.54–1.86	*0.989*
T												
T1	1											
T2	2.08	0.26–16.63	*0.491*									
T3	4.25	0.59–30.76	*0.152*									
T4	8.08	1.03–63.13	*0.046*									
N												
N0	1											
N1	1.2	0.67–2.15	*0.546*									
N2	3.68	2.04–6.63	*0*									
No lymph nodes yield												
≥ 12	1			1			1			1		
< 12	1.6	0.84–3.06	*0.154*	1.94	0.97–3.91	*0.063*	1.65	0.88–3.07	*0.116*	1.95	0.99–3.84	*0.052*
Stage												
Stage I	1			1			1			1		
Stage II	2.35	0.97–5.70	*0.058*	2.70	1.10–6.62	*0.03*	2.09	0.91–4.80	*0.08*	2.41	1.04–5.59	*0.041*
Stage III	3.4	1.43–8.13	*0.006*	3.66	1.48–9.05	*0.005*	3.02	1.34–6.80	*0.008*	3.27	1.40–7.64	*0.006*
Perineural invasion												
No	1			1			1			1		
Yes	1.71	0.78–3.77	*0.18*	0.61	0.24–1.54	*0.297*	1.66	0.76–3.65	*0.204*	0.59	0.24–1.49	*0.266*
Vascular invasion												
No	1			1			1			1		
Yes	2.51	1.42–4.43	*0.001*	2.36	1.17–4.73	*0.016*	2.45	1.39–4.31	*0.002*	2.29	1.15–4.57	*0.019*
Lymphatic invasion												
No	1			1			1			1		
Yes	2.15	1.19–3.89	*0.011*	1.41	0.69–2.89	*0.344*	2.23	1.26–3.96	*0.006*	1.54	0.77–3.08	*0.217*

### Oncologic outcome for stage II patients

Noteworthy, stage II was found as independently related to the DFS (*p = 0.029*) but it did not reach statistically significance regarding recurrence, 5-year survival and OS. Hence, a multivariate analysis among factors which could affect outcome in the 126 stage II patients was conducted (Table 6), disclosing NLR > 4.7 as an independent dismal factor for DFS, 5-year survival and OS, but not for the recurrence itself. After adjusting stage for gender, age, location of the primary tumor, differentiation, as well as the presence of perineural, vascular, and lymphovascular invasion, the significance of NLR > 4.7 became more prominent for DFS, 5-year survival and OS in stage II patients (Table 7).

**Table 6:** Multivariate Cox models among factors which might affect the recurrence, the disease free survival (DFS), the 5-year survival and the overall survival (OS) in stage II patients

	Recurrence			DFS			5-year survival			Overall Survival		
	HR	95% CI	*p value*	HR	95% CI	*p value*	HR	95% CI	*p value*	HR	95% CI	*p value*
NLR												
≤ 4.7	1			1			1			1		
> 4.7	1.68	0.37–7.71	*0.506*	2.76	1.07–7.13	*0.036*	3.84	1.39–10.63	*0.01*	3.62	1.33–9.82	*0.012*
Gender												
Female	1			1			1			1		
Male	1.44	0.44–4.71	*0.546*	1.32	0.63–2.77	*0.464*	1.02	0.44–2.37	*0.961*	1.09	0.48–2.50	*0.838*
Age												
≤ 72	1			1			1			1		
> 72	0.88	0.30–2.57	*0.811*	1.64	0.79–3.41	*0.183*	2.33	0.94–5.75	*0.066*	2.56	1.05–6.26	*0.039*
Primary tumor												
Right colon	1			1			1			1		
Left colon	0.96	0.21–4.40	*0.958*	0.88	0.33–2.35	*0.794*	0.95	0.31–2.94	*0.935*	0.96	0.31–2.95	*0.947*
Rectum	1.98	0.53–7.41	*0.312*	1.75	0.70–4.39	*0.233*	1.59	0.53–4.76	*0.41*	1.74	0.59–5.11	*0.317*
Grade												
Low	1			1			1			1		
Medium + High	1.67	0.37–7.58	*0.506*	1.34	0.53–3.40	*0.541*	1.31	0.45–3.80	*0.625*	1.36	0.47–3.93	*0.569*
No lymph nodes yield												
≥ 12				1			1			1		
< 12				1.82	0.66–5.07	*0.249*	2.76	0.90–8.45	*0.075*	2.4	0.80–7.19	*0.117*
Perineural invasion												
No				1			1					
Yes				0.49	0.08–2.85	*0.426*	0.57	0.09–3.78	*0.562*	0.63	0.10–4.04	*0.624*
Vascular invasion												
No	1			1			1			1		
Yes	2.65	0.53–13.18	*0.234*	2.09	0.71–6.13	*0.182*	2.28	0.66–7.89	*0.192*	2.22	0.65–7.58	*0.205*
Lymphatic invasion												
No	1			1			1			1		
Yes	0.93	0.10–8.70	*0.953*	2.3	0.75–7.02	*0.145*	2.06	0.55–7.69	*0.284*	2.12	0.58–7.80	*0.257*

**Table 7:** Correlation between NLR and DFS, 5-year survival and OS by stage, after adjusting it by gender, age, location of the primary tumor, differentiation as well as presence of perineural, vascular, and lymphovascular invasion

	DFS			5-year survival			Overall Survival		
	HR	95% Confidence Interval	*p value*	HR	95% Confidence Interval	*p value*	HR	95% Confidence Interval	*p value*
Stage I									
NLR > 4.7	1.60	0.19–13.41	*0.663*	2.06	0.23–18.18	*0.514*	1.85	0.22–15.88	*0.572*
Stage II									
NLR > 4.7	2.85	1.21–6.73	*0.017*	4.06	1.66–9.93	*0.002*	4.07	1.67–9.91	*0.002*
Stage III									
NLR > 4.7	1.33	0.54–3.28	*0.537*	1.28	0.50–3.22	*0.605*	1.27	0.50–3.18	*0.612*

## Discussion

Systematic review [32] and meta-analyses [33–37] agreed that elevated pre-treatment NLR predicts poor prognosis in CRC patients, both in those with localized disease as well as in those with liver metastases.

The exact NLR cut-off value varies among the studies. Among the 19 studies included in a systematic review, in one the cut-off value was set at 4.98 and in eight at 5 [32]. In eight out of 13 (61.5%) studies included in a meta-analysis, the cut-off value had been set at 5 [33], in 11 out of 16 (69%) studies included in another meta-analysis, the cut-off value had also been set at 5 [35], a third meta-analysis which included 15 studies, disclosed that patients with pretreatment NLR < 5 were significantly more likely to have 5-year overall survival and 5-year disease-free survival [36], while in the most recent meta-analysis, in 13 out of 16 (81%) included studies, the cut-off value had been set at 5 [37]. These findings suggest that a value close to 5 seems to provide the most statistically significant results.

In agreement to previous reports, the present study concluded that in patients with localized CRC, an NLR above 4.7 was a dismal prognostic factor for DFS, 5-year survival and overall survival.

However, the exact mechanism explaining the adverse association between NLR and survival outcome in CRC patients, remains unknown. Neutrophilia is a common finding in cancer patients. Colorectal cancer cells secrete granulocyte colony stimulating factor which recruits neutrophils into the tumor site [38]; several among the micro-environment cells produce mediators capable of recruiting different leukocytes populations from circulation into the tumor site [4]; while others are capable of secreting neutrophil chemotactic substances [5]. Neutrophils are the primary source of vascular endothelial growth factor [39], while neutrophils-derived proteinases degrade cytokines and chemokines [40, 41] and remodel the extracellular matrix [42], favoring tumor proliferation, local invasion, angiogenesis and tumor vascularization, promoting metastatic potential [39–43]. Moreover, neutrophil elastase, upon gaining entry to the tumor cells, leads to hyperactivity of the PI3K pathway, accelerating the uncontrolled tumor cell proliferation further [5, 44]. Finally, neutrophils are also capable to degrade basement membrane, mediating local tumor invasion and distant metastases formation [45]. Therefore, increased neutrophils may promote tumor growth and metastasis.

The adaptive immune system is mainly represented by tumor infiltrating lymphocytes comprising CD8+ cytotoxic T-lymphocytes and CD4+ T-helper lymphocytes [46]. Gene profiling analyses of tumor microenvironment in a variety of solid tumors revealed that the majority of them showed a T cell–infiltrated phenotype [47]. During the tumor specific adaptive response, cytotoxic T lymphocytes play a crucial role, inducing production of cytokines [48]. Especially cytokine IFN-γ has a pivotal antitumor role inducing cell cycle arrest and proliferation [34], autophagy-associated apoptosis [49] and antitumor macrophage activity [50]. Noteworthy, neutrophils isolated from early-stage, small-sized tumors are able to stimulate T-cell responses and are cytotoxic to cancer cells [51]. Thus, an increased intratumoral lymphocyte concentration may amplify host systemic inflammatory response to a tumor, fact probably associated with a positive clinical outcome [52, 53]. Pre-treatment lymphocytopenia has been proposed as a surrogate marker of cancer-induced immunosuppression. Various immunosuppressive molecules triggered by activated signaling pathways (STAT, MARK, NF-kB, Wnt/β-catenin) as a result of gene alterations [54] or an inherited T cell triggered adaptive resistance [55], impair activation of helper lymphocytes, promote recruitment of suppressive regulatory T cells and activate the extrinsic pathway of apoptosis, finally impairing lymphocytes homeostasis [56].

An elevated NLR can be the result of either an increase in nominator (neutrophils) or a decrease in denominator (lymphocytes) or both. In tumor microenvironment, an increased neutrophils concentration promotes tumor growth, while a decreased lymphocytes concentration indicates ineffective local tumor control. Thus, an increased microenvironmental NLR may indicate tumor progression, representing a marker of dismal prognosis. Since, all published so far reports, unanimously agree that a high serum NLR is an indicator of unfavorable prognosis, we can postulate that the serum NLR reflects indirectly but accurately the intratumoral inflammation process. Since calculation of intratumoral NLR is neither available in all institutes nor cost-effective, while serum NLR is an easily measured, reproducible and cost-effective marker, serum NLR may hold a great clinical impact in the future.

The present study disclosed that the lowest median NLR value was noticed in T1 tumors (1.88) and was gradually increased up to 2.76 in T4 tumors. Even higher median NLR values were noticed among patients who recurred (2.79), particularly among them who developed distant metastases (3.05). Previous reports [57–59] addressed that the lowest median NLR value had been noticed in normal mucosa, increasing gradually in the pathway extending from adenoma to cancer. Therefore, we can propose that host immune system responses with its maximum force at the earliest stage of carcinogenesis, even at the level of precancerous condition, in an attempt to confine tumor locally, subsequently eliminating as cancer cells progressively escape from host immunological surveillance.

In our study, the highest median NLR value was noticed among tumors positive for perineural invasion (3.45), although neural invasion was not disclosed as independent prognostic marker in our multivariate analysis. Perineural invasion is introduced in the seventh edition of TNM [31], as an accessory factor, for poor prognosis. It is believed that perineural invasion is an alternative route of metastatic spread, and it has been associated with poor differentiation, T stage, incidence of metastasis at time of diagnosis, lymphatic, venous invasion and local recurrence. Perineural invasion was also reported to be an independent prognostic factor for 5-year survival and 5-year DFS [60, 61]. We believed that NLR was higher in tumors with perineural invasion as both factors are markers of aggressive phenotype of CRC.

The finding that NLR > 4.7 was directly related to the advanced age of the patients (particularly those over 72 years old) is in agreement to published reports that aging cells create a inflammatory micro-environment more permissive to tumor growth [62].

NLR > 4.7 was found unrelated to the local or distant recurrence, although we tried different cut off values searching for any statistical significance. Cancer cells actively develop different mechanisms to escape tumor immunity. Cancer cells utilize chemokines, which are up-regulated as cancer becomes more malignant, and are key players in cancer cell proliferation and invasiveness, promoting cancer cell metastasis [63]. In response to specific chemokines, different immune cell subsets migrate into the tumor microenvironment and regulate tumor immune responses. Direct and indirect interactions on chemokine pathways may reshape the immune and biological phenotypes of a tumor, making its biological behavior unpredictable and altering its metastatic potential [64].

Even thought NLR > 4.7 was not associated with recurrence, as already stated, high NLR was associated with DFS, this inconsistency in our results, can be explain from the fact that DFS is actually a composite event consisting of either survival or recurrence and the number of recurrent events is low compared to the number of deaths.

The most interesting finding of the present study was that NLR > 4.7 disclosed as an independent dismal prognostic factor for DFS, 5-year survival and overall survival in stage II CRC patients. After adjusting stage for gender, age, location of the primary tumor, differentiation, and presence of perineural, vascular and lymphovascular invasion, NLR > 4.7 was isolated as dismal prognostic factor only for stage II patients.

According to the American Joint Committee on Cancer (AJCC), stage II CRC includes three subcategories: stage IIA (T3 N0), stage IIB (T4aN0) and stage IIC (T4bN0). Seventy five percent of stage II CRC patients can be cured with surgery alone, not experiencing any further recurrence, while the remaining 25% will develop recurrence in the future [65]. The definition of whom among the CRC stage II patients constitute a ‘high-risk’ subpopulation, which will be favored by adjuvant chemotherapy is not clearly defined in the TNM staging system and there is no clear consensus in the literature [66].

European Society for Medical Oncology (ESMO) recommends adjuvant chemotherapy for those individuals who fulfill at least one of the following criteria: T4 tumors, poorly differentiated tumors, tumors with vascular, lymphatic or perineural invasion, inadequate sample of lymph nodes (< 12) and bowel perforation or bowel obstruction at presentation [67].

The National Comprehensive Cancer Network (NCCN) furthermore recommends adjuvant chemotherapy for patients with inadequate or positive resection margins, with tumors characterized as MSI-L or MSS, with no significant comorbidities and anticipated life expectancy [66]. Stage II MSI-H CRC tumors do not benefit from adjuvant therapy [68].

The prognostic significance of NLR has also been examined in the subgroup of stage II CRC patients. Ding et al [69] disclosed that an elevated NLR was related to a worse 5-year survival. Hung et al [70] addressed that an elevated NLR was an independent predictor for OS but not for DFS because the patients of his study with an elevated NLR tended to have an increased risk of death from other causes and an elevated NLR was linked with some stronger risk factors such as T4b cancers, tumor obstruction or tumor perforation. Two reports from the same Institute [71, 72], concluded that an elevated NLR was related to a decreased OS and a decreased time-to-relapse, particularly in the group of patients who underwent curative surgery alone compared to them who underwent adjuvant chemotherapy. The authors concluded that an elevated NLR may be a negative prognostic marker, and that such high-risk patients may benefit from adjuvant chemotherapy. The most recent report is coming from Turner et al [15] who found that the combination of low intratumoral chronic inflammatory cells density with high serum NLR level, served a poor outcome in terms of recurrence-free survival and OS for stage II CRC patients, finally suggesting that in this particular subgroup of patients, adjuvant chemotherapy might be considered.

After adjusting stage by gender, age, location of the primary tumor, differentiation, the presence of perineural, vascular and lymphovascular invasion, the present study isolated NLR > 4.7 as an independent dismal prognostic factor only for stage II CRC patients. We hypothesize that in stage III patients, the well-known prognostication of the metastatically infiltrated lymph nodes [73, 74] represents the strongest factor finally defining the poorer outcome. The new finding that in stage II CRC patients an elevated NLR may be by itself an independent dismal prognostic factor should be evaluated further in order to be determined its prognostic significance (if any) and its possible clinical implications.

## Conclusion

The present study concluded that in patients with localized CRC, a pretreatment NLR above 4.7 is a dismal prognostic factor for disease free survival (DFS), 5-year survival and overall survival (OS). The dismal prognostic effect of NRL is magnified in Stage II CRC patients.

## Abbreviations

AJCC: American Joint Committee on Cancer
CI: Confidence Interval
CRC: Colorectal Cancer
CRP: C-reactive protein
CT: Computer tomography
DFS: Disease Free Survival
ESMO: European Society for Medical Oncology
HR: Hazard Ratio
INF-γ: Interferon gamma
IR: Interquartile Range
MMR: Mismatch repair
MRI: Magnetic resonance imaging
MSI: Microsatellite instability
NCCN: National Comprehensive Cancer Network
NLR: Neutrophil to lymphocyte ratio
OS: Overall Survival
ROC: Receiver Operating Characteristic
TNM: TNM Classification of Malignant Tumors
